# Highly Antifungal Activity of Biosynthesized Copper Oxide Nanoparticles against *Candida albicans*

**DOI:** 10.3390/nano12213856

**Published:** 2022-11-01

**Authors:** Luis Enrique Garcia-Marin, Karla Juarez-Moreno, Alfredo Rafael Vilchis-Nestor, Ernestina Castro-Longoria

**Affiliations:** 1 Department of Microbiology, Center for Scientific Research and Higher Education of Ensenada (CICESE), Carr. Tijuana-Ensenada 3918, Zona Playitas, Ensenada 22860, Baja California, Mexico; 2 Center for Applied Physics and Advanced Technology, UNAM, Blvd. Juriquilla 3001, Juriquilla La Mesa, Juriquilla 76230, Queretaro, Mexico; 3 Sustainable Chemistry Research Joint Center UAEM—UNAM (CCIQS) Toluca-Atlacomulco Road Km 14.5, San Cayetano 50200, Toluca, Mexico

**Keywords:** copper oxide nanoparticles, green synthesis, antifungal inhibition, cytotoxicity

## Abstract

*Candida albicans* (ATCC SC5314) was exposed to biosynthesized copper oxide nanoparticles (CuONPs) to determine their inhibitory capacity. Nanoparticles were polydisperse of small size (5.8 ± 3.5 nm) with irregular shape. The minimum inhibitory concentration (MIC) against *C. albicans* was 35.5 µg/mL. The production of reactive oxygen species (ROS) of *C. albicans* was verified when exposed to different concentrations of CuONPs. Ultrastructural analysis of *C. albicans* revealed a high concentration of CuONPs in the cytoplasm and outside the cell; also, nanoparticles were detected within the cell wall. Cytotoxic analyses using fibroblasts (L929), macrophages (RAW 264.7), and breast (MCF-12) cell lines show good results of cell viability when exposed at the MIC. Additionally, a hemocompatibility analysis was carried out and was found to be below 5%, considered the threshold for biocompatibility. Therefore, it is concluded that the biosynthesized CuONPs have a high potential for developing a topical antifungal treatment.

## 1. Introduction

Candidiasis is caused by different *Candida* species, with *C. albicans* being the main cause of the disease worldwide [1]. There are several important species of *Candida*, which tend to be infectious for humans, among these *C. tropicalis*, *C. glabrata*, *C. parapsilosis*, *C. stellatoidea*, *C. krusei,* and *C. kyfer*. In the last 30 years, *C. glabrata*, *C parapsilosis,* and *C. tropicalis* have considerably increased the cases of candidiasis [2], but *C. albicans* is still the most frequently isolated from patients [3]. Unfortunately, there are increasing reports of *Candida* species with multidrug resistance to conventional antifungals, such as *C. glabrata* and, more recently, *Candida auris* showing resistance to the azoles, echinocandins, and polyenes [4]. Therefore, it is necessary to look for new treatments to combat the resistance of these microorganisms. Nanotechnology offers novel alternatives to combat pathogenic microorganisms since metallic nanoparticles such as silver, copper, and gold, among others, have antimicrobial properties [5].

Various studies have shown the antifungal properties of metallic nanoparticles, including silver nanoparticles (AgNPs), to which *C. albicans* is susceptible [6,7,8]. Inhibition of the fungus has been reported at low concentrations, preventing its growth and affecting mature biofilm formation [6]. Additionally, combining AgNPs with antifungals such as fluconazole (FLZ) increases their inhibitory capacity. These results are key to exploring their clinical use since the amount of antifungal and/or nanomaterials can be reduced, and thus, secondary or toxic effects caused by any of these two components can be minimized [8].

On the other hand, the antimicrobial activity of copper-based nanoparticles has not been fully studied, but the antimicrobial effectiveness of chemically synthesized copper nanoparticles has also been reported, including against *C. albicans* [9]. However, chemical synthesis could contain toxic residues, and then, green synthesis can be a better option for biomedical applications such as their use as antimicrobials [5]. In fact, a clear advantage has been demonstrated in using biosynthesized copper nanoparticles against *C. albicans* [10]. Among the biological materials utilized for biosynthesis, fungi have great potential for the manufacture of nanoparticles since they have the ability to secrete large amounts of enzymes, metabolites, etc., in addition to their easy handling in the laboratory [11]. Fungi have certain advantages for synthesizing nanoparticles, unlike other organisms such as bacteria or plants, as the fungal mycelium can withstand flow pressure, agitation, and different factors on bioreactors compared to other organisms used. The most used fungi for the biosynthesis of nanoparticles are *Fusarium* spp., *Aspergillus* spp., *Penicillium* spp., and *Trichoderma* spp. [12]. In this work, we screened several species of *Trichoderma* to produce copper oxide nanoparticles (CuONPs) with antifungal activity. The disk diffusion method was used as a first approach, and the CuONPs with the highest antifungal activity were chosen to evaluate their potential use against *C. albicans*. The CuONPs obtained displayed excellent antifungal activity, and also the minimum inhibitory concentration (MIC) used for *Candida* resulted in a non-toxic concentration for three mammalian cell lines, then the medical application of these nanoparticles is promising.

## 2. Materials and Methods

### 2.1. Strain, Media, and Growth Conditions

*Candida albicans* (ATCC SC5314 strain) was cultured at 37 °C in liquid YPD media (1% yeast extract, 2% peptone, 2% dextrose). Additionally, solid YPD agar plates were used (2% bacteriological agar added). Several *Trichoderma* strains were cultured to obtain the supernatant to be used as a reducing agent. Liquid cultures were prepared by adding 25 µL of *Trichoderma* spores at a concentration of 1 × 10^6^ in an Erlenmeyer flask with 250 mL of PDB and incubated for three days at 30 °C, with agitation at 120 rpm. Biomass was obtained by filtration and washed three times with distilled water, and incubated for three additional days in deionized water at 30 °C. After incubation, the supernatant was obtained and filtered with a 0.22 μm nitrocellulose membrane and used immediately or kept at 4 °C until use.

### 2.2. Biosynthesis of CuONPs

Copper oxide nanoparticles (CuONPs) were synthesized using a solution of pentahydrated copper sulfate (CuSO_4_.5H_2_O) (Sigma-Aldrich, St. Louis, MI, USA). The solution was mixed in various proportions (1:1, 1:2, 1:3, reducing agent/copper sulfate) with the supernatant of several *Trichoderma* strains, which were used as reducing agents. NaOH was used to adjust the pH, and the solution was incubated for 24 h at room temperature (24–25 °C). Several reactions were carried out until the nanoparticles were obtained; the color change of the reaction is evidence of nanoparticle formation. Nanoparticles were first screened for antifungal activity by the disk diffusion method, and those with the highest antifungal activity were selected for this study (Patent pending).

### 2.3. Characterization of CuONPs

Synthesized nanoparticles were first evaluated by UV-Vis spectroscopy at 200 to 700 nm after 24 h of incubation; at this time, the color of the reaction changed from clear to pale blue. A Perkin Elmer precisely UV-Vis lambda/25 spectrophotometer was used (PerkinElmer Inc., Waltham, MA, USA). The zeta potential and the hydrodynamic diameter (HD) of synthesized CuONPs were measured with a Zetasizer Nano ZS instrument (Malvern Panalytical Inc., Westborough, MA, USA).

The size and shape of CuONPs were analyzed under transmission electron microscopy (TEM) (Hitachi H7500, Hitachi Ltd., Tokyo, Japan) at 100 kV. Additionally, samples were placed on formvar/carbon-coated nickel grids of 400 mesh and analyzed under high-resolution transmission electron microscopy (HRTEM) (JEM-2100 from JEOL, JEOL Ltd., Tokyo, Japan) operated at 200 kV to study the size, morphology and the crystal lattice of the particles. The size of nanoparticles was determined using the ImageJ program (free version for Windows 1.8.0_172). The OriginPro 2021 version 9.8.0.200 program was used for statistical analyses.

### 2.4. FTIR Analysis of Synthesized CuONPs

To determine the functional groups from the reducing agent attached to nanoparticles, a freshly prepared sample of CuONPs was placed on a glass slide and allowed to dry at room temperature. Afterward, the Fourier transform infrared spectroscopy (FTIR) spectrum was collected using a Bruker Tensor 27 FT-IR spectrometer (SpectraLab Scientific Inc., Markham, ON, Canada) with a resolution of 0.5 cm^−1^ in transmission mode. An average of 5 scans for the spectrum was recorded over the 500–4000 cm^−1^ wavelength range.

### 2.5. Evaluation of Antifungal Capacity

The broth microdilution method was used to obtain the minimum inhibitory concentration (MIC) according to the CLSI document M27-A2 for yeast [13], with slight modifications. In a 96-well microplate, 100 µL of RPMI medium was inoculated with *C. albicans* ATCC SC5314 at a concentration of 1.5 × 10^5^, and then different concentrations of CuONPs were added. The concentrations of CuONPs were: 22.2, 44.4, 66.6, 88.8, and 111.1 µg/mL. Afterward, to define a more specific MIC, concentrations tested were: 22.2, 24.4, 26.6, 28.8, 31.0, 33.3, 35.5, 37.7, 39.9, 42.1, and 44.4 µg/mL. Concentrations were placed in ascending order, and all wells were filled to 200 µL with cell-free RPMI medium. The negative control contained only cells without any treatment, and another well contained only RPMI medium to verify the sterility of the experiment. The plate was incubated at 37 °C, and observations were made at 24 and 48 h; the experiment was performed in triplicate. After 48 h of incubation, 10 µL was taken from each well and inoculated in Petri dishes with YPDA medium. Optical density was recorded at an absorbance of 550 nm. It was taken as 100% of cell viability, the negative control. Afterward, the antifungal capacity of CuONPs was tested by the diffusion disk method and compared with conventional antifungals. The disk diffusion method was performed using the protocol of document M44-A for yeasts of the Clinical Laboratory Standards Institute [14].

### 2.6. ROS Production

Production of reactive oxygen species (ROS) was carried out as follows: *C. albicans* cells were inoculated in a 96-well microplate at a concentration of 1.5 × 10^5^, at 37 °C for 24 h with different concentrations of CuONPs, which were placed in ascending order, 33.3, 35.5, 37.7, 39.9, and 42.1 µg/mL, a well was left as a negative control only with cells without treatment. After centrifugation at 1500 rpm for 10 min, the supernatant was discarded, and PBS was added; this step was performed three times. Afterward, 200 µL of PBS and DCFDA (20,70-dichlorofluorescein diacetate, 45 µM) were added to all wells, and the microplate was incubated for 60 min at 37 °C. Finally, fluorescence was measured with a Cary Eclipse fluorescence spectrophotometer (Agilent Technologies, Santa Clara, CA, USA) using an excitation and emission wavelength of 485 and 530 nm, respectively.

### 2.7. Ultrastructural Analysis of C. albicans

To determine CuONPs internalization, *C. albicans* was grown in liquid cultures with CuONPs at the MIC of 35.5 µg/mL. After 24 h, cells were fixed with 2.5% glutaraldehyde in 0.05 M sodium phosphate for 30 min. at ambient temperature. Subsequently, cells were post-fixed with 2% OsO_4_ at 4 °C for 2 h. Afterward, cells were dehydrated in ethanol series and infiltrated in resin/ethanol series, according to Vazquez-Muñoz et al. [8]. After polymerization, samples were mounted in resin blocks and sectioned in a Leica Ultracut-R ultramicrotome (Leica Microsystems Inc., Buffalo Grove, IL, USA). Sections 70 nm thick were mounted in formvar/carbon 75 mesh copper grids and analyzed under TEM (Hitachi H7500), operated at 100 keV and spot size 5. Samples were not post-stained for better CuONPs detection.

### 2.8. Biocompatibility Evaluation

#### 2.8.1. Cell Viability

To evaluate the biocompatibility of the biosynthesized CuONPs, cell viability tests were assessed with different cell lines, fibroblasts (L929), macrophages (RAW 264.7), and breast cells (MCF-12F). The protocol to evaluate cytotoxicity was the metabolic reduction of the bromide compound 3-(4,5-dimethylthiazol-2-yl)-2,5-diphenyltetrazole (MTT). Cell lines were cultivated in Dulbecco’s modified Eagle medium (DMEM), which was supplemented with 1% L-glutamine, 1% antibiotic/antifungal, 10% fetal bovine serum, and 1.5 g sodium bicarbonate. Cells grown in DMEM without CuONPs were used as a negative control. Cells were exposed to different concentrations of CuONPs, according to those used in the analysis, to obtain the MIC: 33.3, 35.5, 37.7, 39.9, and 42.1 ug/mL. Additionally, the reducing agent used for the synthesis of CuONPs was evaluated. The assays were as follows: 10,000 cells per well were seeded in a 96-well microplate and incubated for 24 h at 37 °C in an atmosphere of 5% CO_2_, then the different concentrations of CuONPs and the reducing agent were added, leaving a final volume of 100 µL per well, samples were incubated for 24 h at 37 °C in an atmosphere of 5% CO_2_. After incubation, samples were washed three times with PBS, adding 200 µL. Subsequently, 10 µL of MTT (0.25 mg/mL) and 90 µL of DMEM were added to each well, and finally, the plates were incubated for 4 h at 37 °C in an atmosphere of 5% CO_2_. Finally, 100 µL of isopropanol was added to dissolve the formazan crystals formed by the reduction of MTT; absorbance was measured at 570 and 690 nm. The results were taken by adjusting the negative control to 100% viability. Each assay was performed in triplicate.

#### 2.8.2. Hemolysis

The hemolysis assay was carried out according to ISO 10993-4 [15]. Briefly, 270 µL of erythrocytes were placed in a 96-well plate, and treatment with CuONPs was used at different concentrations (33.3, 35.52, 37.74, 39.96, and 42.18 µg/mL). The final volume was completed to 300 µL with DMEM medium, no treatment was added to the negative control, and 30 µL of Triton-100 at 20% was used for the positive control. The plate was left to incubate for one hour at 37 °C and 5% of CO_2_. Afterward, the plate was centrifuged for 5 min at 3000 rpm, the resulting supernatant was transferred to another 96-well microplate, and the absorbance at 541 nm was measured in a UV-visible spectrophotometer (Thermo Scientific Multiskan GO, Vantaa, Finland).

## 3. Results

### 3.1. Nanoparticle Characterization

**Figure 1:**
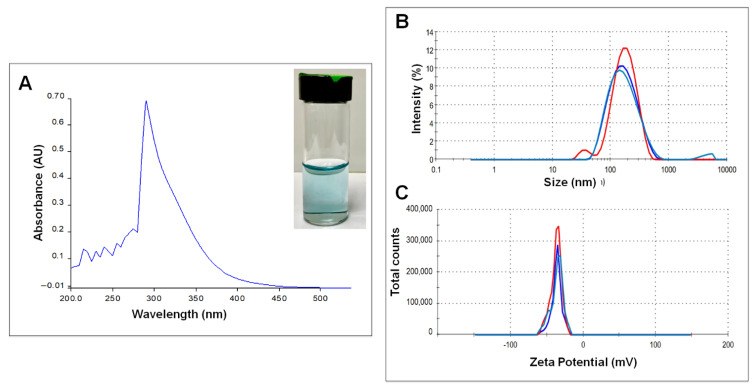
(**A**) Absorbance curve of copper oxide nanoparticles, (**B**) hydrodynamic diameter, (**C**) Zeta Potential. The inset in (**A**) shows the newly synthesized CuONPs.

During the synthesis process, the formation of CuONPs was evident when the solution turned from clear to light blue (Figure 1A inset). The biosynthesized CuONPs showed an absorbance peak at 290 nm in UV-visible spectroscopy, which is attributed to the formation of oxide copper nanoparticles [16] (Figure 1A). The hydrodynamic size and surface charge of the CuONPs were determined using dynamic light scattering (DLS) analysis. The hydrodynamic size of the CuONPs was 143.3 nm (Figure 1B), and the Z potential was −32.6 mV (Figure 1C). 

**Figure 2:**
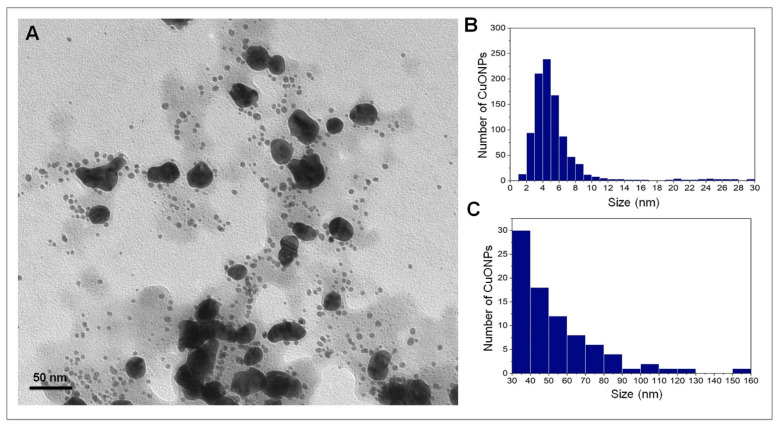
(**A**) Micrograph of the copper oxide nanoparticles synthesized by a biological method; (**B**) Histogram of the smallest nanoparticles; (**C**) Histogram of the largest nanoparticles. *n* = 1000.

Nanoparticles were analyzed by TEM for shape and size examination, and it was determined that CuONPs are polydisperse; 1000 nanoparticles were measured, and two size populations were found (Figure 2A); the vast majority were quasi-spherical with sizes ranging from 1.3 to 30 nm (Figure 2B), with an average size of 5.8 ± 4.8 nm. The second population of NPs (8.4%) presented a variable morphology (Figure 2A) and a size ranging from 30 to 160 nm (Figure 2C), with an average size of 54.1 ± 23.5 nm.

**Figure 3:**
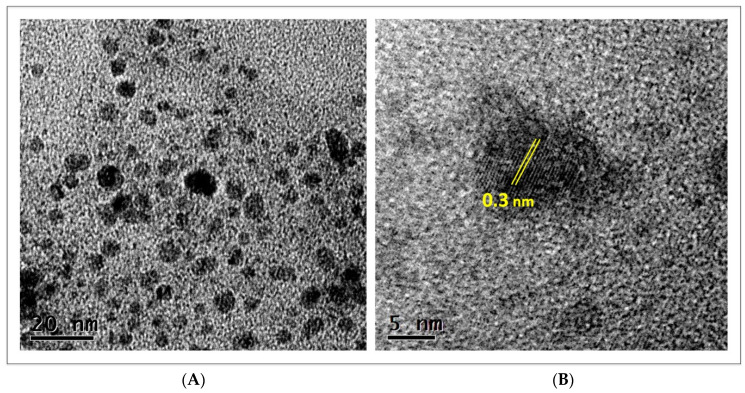
Micrographs of high-resolution transmission electron microscopy of CuONPs synthesized by a biological method; (**A**) image showing polydispersity and variable shape of nanoparticles; (**B**) lattice fringe space of nanoparticles.

Nanoparticles were also analyzed by HRTEM; in Figure 3, representative images of CuONPs are presented. At higher resolution, it becomes clearer that small nanoparticles are very abundant and have quasi-spherical or irregular shapes (Figure 3A). In the analysis of a single particle, the crystalline nature of nanoparticles is confirmed, with the lattice fringes spacing about 0.3 nm (Figure 3B). 

### 3.2. FTIR Analysis of CuONPs

**Figure 4:**
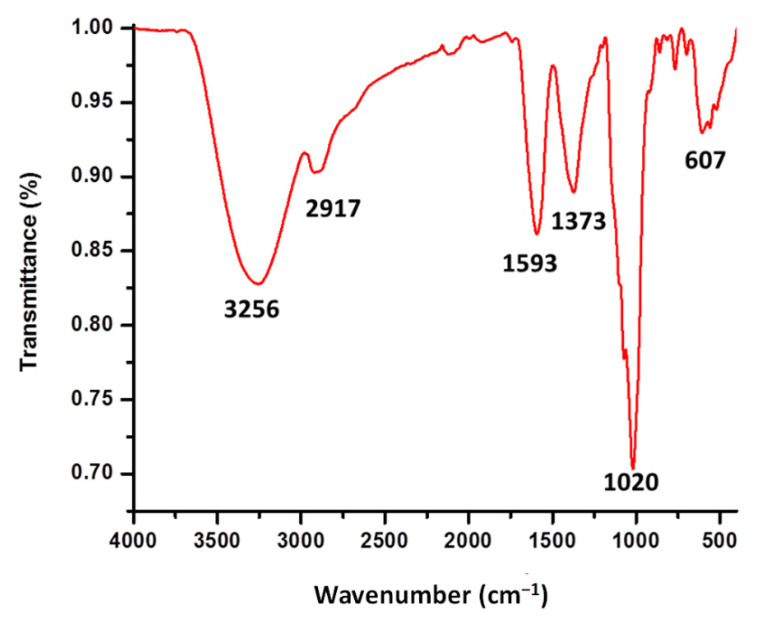
FTIR spectra of synthesized CuONPs.

Analysis of CuONPs Fourier transform infrared spectroscopy (FTIR) was carried out to elucidate the functional groups involved in the reduction and stabilization of nanoparticles. Two primary bands were revealed, 3256 and 1020 cm^−1^, and four secondary bands, 2917, 1593, 1373, and 607 cm^−1^ (Figure 4). The band 3256 cm^−1^ corresponds to the N–H and O–H stretching vibrations indicating primary and secondary amines. The band 1020 cm^−1^ corresponds to the C–O bending and C–N stretching of aliphatic amines. The band 2917 cm^−1^ corresponds to the C–H (aliphatic) stretching vibrations, indicating stretching in methyl groups; the peak at 1593 corresponds to the C=C aromatic ring system accompanied by N-H bending, the peak at 1373 corresponds to the –C–N stretching vibrations and the 607 cm^−1^, generally C-X bends can be found, which indicates the presence of organohalogens. 

### 3.3. Evaluation of the Antifungal Properties of CuONPs against C. albicans

**Figure 5:**
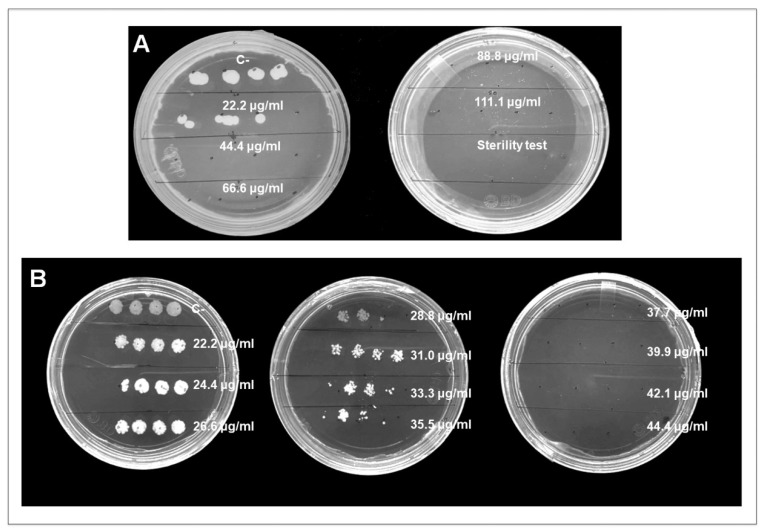
(**A**) Growth of *C. albicans* after 48 h treatment with CuONPs at different concentrations; (**B**) Colony growth after 48 h of incubation with CuONPs for MIC determination after broth microdilution assay. C- = negative control.

**Figure 6:**
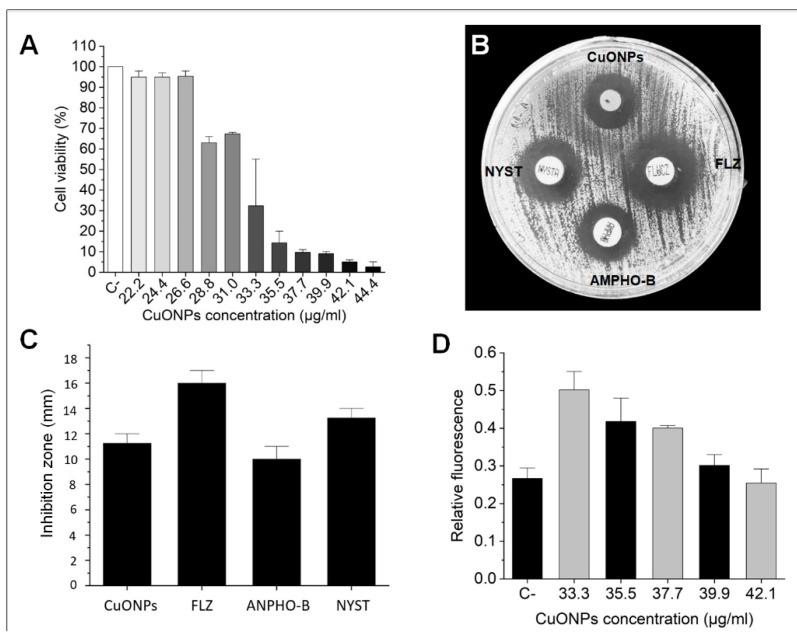
(**A**) Cell viability of *C. albicans* after 48 h of incubation with CuONPs at different concentrations for MIC determination; (**B**) Inhibition of *C. albicans* by CuONPs and conventional antifungals; (**C**) Histogram of inhibition zone diameters obtained by the antifungal susceptibility test after 24 h of incubation; (**D**) ROS production by *C. albicans* after 24 h exposure to CuONPs. C- = negative control; FLZ = Fluconazole; ANPHO-B = Amphotericin B; NYST = Nystatin.

Antifungal activity of the biosynthesized CuONPs was first tested in a broad range, from 22.2 to 111.1 µg/mL (Figure 5A). In this assay, *C. albicans* stopped growing at a concentration of 44.4 µg/mL (Figure 5A). A concentration range of 22.2–44.4 µg/mL was tested to define a more specific MIC. The results obtained showed that *C. albicans* grows similarly to the negative control at concentrations of 22.2, 24.4, and 28.8 µg/mL (Figure 5B). At higher concentrations, growth decreases with a MIC at 35.5 µg/mL (Figure 6A). At concentrations of 39.9–44.4 µg/mL, growth is completely inhibited (Figure 5B), and no growth was detected for five consecutive days. 

The disk diffusion method verified the antifungal activity of the minimum inhibitory concentration (MIC) of CuONPs. *C. albicans* was susceptible to the conventional antifungals at the prescribed concentrations; Fluconazole (25 µg), Amphotericin-B (10 µg), and Nystatin (50 µg) (CLSI) [14] (Figure 6B). It was interesting to note that a larger inhibition diameter by the CuONPs was observed during the first 10 h, but *Candida* was able to grow within this zone but not in the zone closer to the disc, defining a clearer inhibition zone (Figure 6B). The antifungal activity of CuONPs at a concentration of 35.5 µg/mL showed comparable zones of inhibition to the conventional antifungals; nevertheless, at this concentration, Fluconazole displayed larger zones of inhibition (Figure 6C).

### 3.4. ROS Production

The ROS produced by *C. albicans* can be seen in Figure 6D; the negative control indicates the basal production of ROS from the cells. The treatment at 33.3 µg/mL of CuONPs indicates an increase in ROS production almost double that observed at the basal reading. However, from a concentration of 35.5 µg/mL, the ROS production starts to decrease until reaching values similar to the baseline production (Figure 6D); this downward trend may be due to the high mortality of *C. albicans* caused by the treatment of CuONPs.

### 3.5. Ultrastructural Analysis of C. albicans

**Figure 7:**
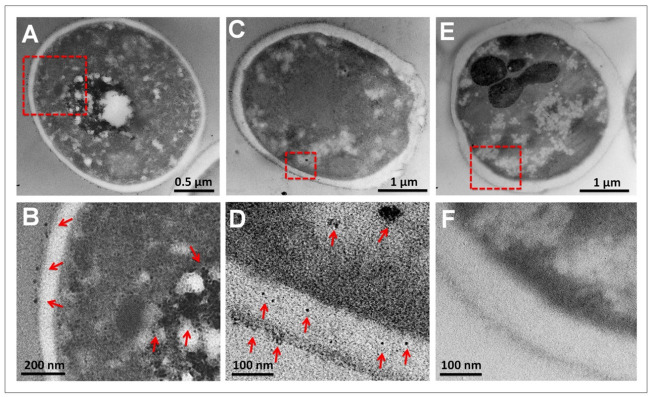
Micrographs of *C. albicans* after incubation for 24 h. (**A**) Cell exposed to CuONPs, showing a high internal accumulation of CuONPs; (**B**) amplification of marked area in (**A**) showing external and internal accumulation of nanoparticles; (**C**) cell exposed to CuONPs showing disrupted cell wall; (**D**) amplification of marked area in (**C**) showing NPs within the cell wall and cytoplasm; (**E**) cell from control culture; (**F**) amplification of marked area in (**E**). Red arrows point out CuONPs.

Liquid cultures of *C. albicans* exposed to CuONPs were analyzed by TEM to detect the interaction of NPs with cells. An accumulation of NPs was observed outside the cell and throughout the cytoplasm (Figure 7) after 24 h of treatment. Due to the small size of NPs (5 nm approx.), their observation at low magnifications was difficult (Figure 7A,C). However, at higher magnifications, the presence of NPs was evident; even after amplifying the micrograph, the accumulation of NPs is clear (Figure 7B,D). Cells exposed to CuONPs were compared with control cells in order to avoid any possible artifact, and no accumulation of any material was observed, as can be seen in Figure 7E,F. No staining or post-staining was used in the samples to prevent accumulation/precipitation of any other metal; only osmium tetroxide was used in the fixation protocol.

### 3.6. Biocompatibility of CuONPs in Mammalian Cell Lines

**Figure 8:**
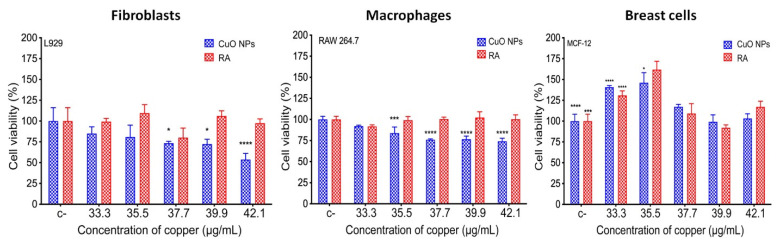
Cell viability assay in fibroblasts (L929), macrophages (RAW 264.7), and breast cells (MCF-12) exposed to biosynthesized CuONPs. The bars represent the mean and standard deviations of experiments performed in triplicate. RA = Biological reducing agent, used for the synthesis of CuONPs. The bars represent the mean and standard deviations of experiments performed in triplicate. * *p* ≤ 0.05, *** *p* ≤ 0.001, and **** *p* ≤ 0.0001.

To evaluate the biocompatibility of CuONPs, three different mammalian cell lines were exposed to several CuONPs concentrations (Figure 8). The concentrations of CuNOPs were selected according to those concentrations at which more than 50% inhibition of *Candida* was detected (33.3–42.1 µg/mL). For fibroblasts (L929), cell viability decreases in a copper-concentration-dependent manner; it can be observed that at concentrations of 33.33 and 35.5 µg/mL, cell viability is above 75%. However, a slightly toxic effect was detected at 37.7–42.1 µg/mL concentrations, where cell viability was below 75%. Macrophages (RAW 264.7) were more tolerant than fibroblasts at the higher concentrations (37.7–42.1 µg/mL), with cell viability near 75%. In the case of breast cells (MCF-12), no cytotoxic effect was detected at any of the analyzed concentrations; furthermore, an increase in cell proliferation was detected at 33.33 and 35.5 µg/mL. It is important to note that all cell lines exposed to the reducing agent alone did not show any cytotoxic effects (Figure 8).

### 3.7. Hemolysis

**Figure 9:**
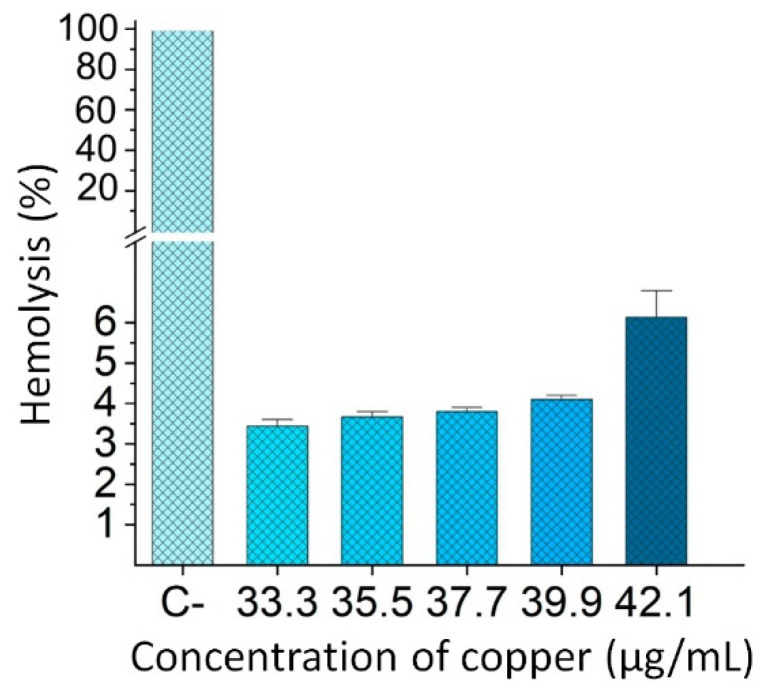
Percentage of hemolysis of red blood cells (RBC) incubated in different concentrations of CuONPs. *n* = 3. Values are the mean ± SD.

To assess the impact of CuONPs on erythrocytes, the hemolysis test was carried out by the spectrophotometric method. The concentrations of CuNOPs were selected according to the concentrations at which more than 50% of *Candida* inhibition was detected (33.3–42.1 µg/mL). As shown in Figure 9, at all concentrations of CuONPs, except at 42.1 µg/mL, hemolysis is below the 5% allowed by ISO 10993-4; therefore, concentrations below 39.9 µg/mL can be considered hemocompatible.

## 4. Discussion

*Candida albicans* is the leading cause of candidiasis disease worldwide, and it is included in the list of microorganisms that present resistance to conventional antifungals [17]. Nanotechnology could be a feasible option to combat resistant microorganisms by using nanoparticles. However, chemicals used to synthesize NPs are potentially toxic to human cells. For this reason, the so-called green synthesis of metallic NPs using different organisms is currently being explored [18]. Copper nanoparticles have great potential in biomedicine due to their antimicrobial, antioxidant, anticancer, and antiviral properties, among others [19,20]. Therefore, exploring the utility of biologically synthesized copper nanoparticles becomes important in searching for biocompatibility. Their great stability indicates they could have a high potential, not only in biomedicine but also in other fields [21]. At present, few studies have explored the antifungal properties of CuONPs, which is important to find new alternatives against strains presenting resistance to conventional antifungals. CuONPs could be a good alternative to treat microbial infections, especially because copper is an essential nutrient for organisms and could have fewer toxic effects than other metallic nanoparticles [22]. For that reason, the aim of this study was to obtain biosynthesized small and stable CuONPs and evaluate their antifungal capacity against the model system *C. albicans*. Additionally, it was important to evaluate their effect in mammalian cell lines and corroborate the safety of CuONPs and the reducing agent used in the biosynthesis process. 

The CuONPs obtained in this study have a small size range and quasi-spherical and/or irregular shape, similar to other biologically synthesized copper nanoparticles [23,24,25], and those using fungal extracts as reducing agents [26,27]. The CuONPs were analyzed by DLS to measure the hydrodynamic diameter (HD) and determine their zeta potential. The HD obtained was 145.3 nm, differing from the size obtained by TEM, which was smaller (5.8 nm). This is because the HD measurement includes the “protein corona” of the nanoparticle, which is constituted by the molecules acting as reducer agents and agglomerate to form this corona [28]. The Z potential of CuONPs was −32.6 mV, and according to the commonly used guidelines, values of ±30 mV are considered highly stable [29].

FTIR analysis provides information on the organic functional groups attached to the nanoparticles; they act as reducing agents for the synthesis and also could serve for their stabilization. The different characteristic peaks of the functional groups were determined according to Socrates [30]. The spectrum obtained for CuONPs was found to be similar to the spectrum reported by different authors [31,32,33]; in all cases, the authors used *Trichoderma harzianum* supernatants as reducing agents. They reported the presence of alcohol, phenols, carbonyl, amines (both aromatic and aliphatic), and amide functional groups.

After obtaining small and stable CuONPs, the antifungal capacity was determined, and the minimum inhibitory concentration (MIC) was 35.5 µg/mL. Although the number of published articles using biosynthesized CuONPs for *C. albicans* inhibition is limited, the CMI found in this study is lower than in other reports. For instance, CuONPs coated with essential oils from *Artemisia absinthium* were reported with a MIC of 500 µg/mL [34]. In another study using the extract of *Prunus mahaleb* as a reducing agent, a MIC of 125 µg/mL was found [35]. The hydroalcoholic extract of *Moringa oleifera* leaves was also used to synthesize copper nanoparticles. The inhibitory capacity against *C. albicans* was reported with a MIC of 62.5 µg/mL [36]. A similar MIC was reported using chemically synthesized CuONPs to inhibit *Candida* (50 µg/mL) [37], close to that obtained in the present study (35.5 ug/mL). However, in the chemical synthesis of CuONPs, there is a generation of residues considered harmful to health and the environment [18]. Considering that the use of metallic nanoparticles has promising applications in biomedicine, it is important to search for efficient NPs to use low concentrations and safe, reducing agents for the synthesis protocol [5].

The disk diffusion method was used to compare the antifungal capacity of the biosynthesized CuONPs (35.5 ug/mL) in relation to the antifungals commonly used to treat candidiasis, such as Fluconazole, Nystatin, and Amphotericin-B. The results obtained in this work show an inhibition diameter of the biosynthesized CuONPs of about 12 mm, greater than that of Amphotericin-B but slightly smaller in comparison with Fluconazole and Nystatin (Figure 6B). The results obtained are considerably good in comparison with the diameter of inhibition of copper nanoparticles synthesized with *Tilia* spp., using 50 µg/mL (7 mm); it was found that inhibition was dose-dependent since an increase to 200 µg/mL increased the inhibition diameter to 14 mm [38]. These concentrations are higher than the MIC used in the present study, and nanoparticles were similar in size, then it is possible that the reducing agent can potentiate the antimicrobial activity of CuONPs.

The antimicrobial activity of nanoparticles causes cellular damage in microorganisms, such as: disrupting membrane functions and thus affecting permeability and respiration, attacking different cell structures, and one of the most important is their ability to alter proteins that contain sulfur and phosphorus; this allows them to directly affect DNA and prevent the development of immune mechanisms [39]. After the exposure of *C. albicans* to different CuONPs concentrations for 24 h, nanoparticles bioaccumulate inside the cells, causing the production of reactive oxygen species (ROS) [37], which damage cellular components such as DNA, protein, and lipids [40], leading to growth arrest and cell death [37,41]. In this work, a decreasing trend of ROS can be observed as the concentration of CuONPs increases; the concentrations closest to the MIC show the highest peak in the production of ROS with respect to the negative control, and as the concentration increases, the production of ROS decreases, this is directly related to the treatment since at concentrations above 37.7 µg/mL a high percentage of mortality in *C. albicans* cells was registered. 

The bioaccumulation of CuONPs in *C. albicans* was investigated by an ultra-structure analysis. In some cases, upon examination of *Candida* cells at low magnifications, it was possible to detect the accumulation of nanoparticles inside the cytoplasm; however, in some cells, it was not so clear the presence of nanoparticles due to their small size. Therefore, it was necessary to analyze the samples at higher amplifications, and the accumulation of CuONPs was clear inside, outside, and within the cell wall. After nanoparticles pass through the cell wall, they interact with the ergosterol, which is an integral component of the *C. albicans* cell membrane that contributes to the permeability, fluidity, and integrity of the cell [42]. Then, if ergosterol is affected, it has a direct consequence on the viability of the fungus. In a recent study, the interaction of copper nanoparticles with *C. albicans* was analyzed, and the results showed that the production of ergosterol decreased, and the morphological switching of yeast-to-hyphae was suppressed by both CuONPs and Cu_2_ONPs. However, the authors found that CuONPs are internalized more efficiently and possess better antifungal activity at lower concentrations than Cu_2_ONPs, and then they suggested the use of small CuONPs for better biological activity [37]. When *C. albicans* is exposed to low concentrations of copper nanoparticles, cells have no significant changes in cell morphology; however, at higher concentrations (518.8 μg/mL), cells display severe damage and changes in their cellular morphology [43]. 

As previously mentioned, copper nanoparticles have great potential use in biomedicine. Therefore, it becomes important to explore their cytotoxicity in different mammalian cell lines. Copper is one of the nutrients humans need for cell functioning. However, exposure to high concentrations of copper nanoparticles, whether through ingestion, inhalation, or constant exposure, can result in adverse and toxic effects [44,45]. To verify the cytotoxicity of CuONPs in the present study, different cell lines were used to determine cell viability using the MTT reduction method. Three different cell lines (fibroblasts L929, macrophages RAW 264.7, and breast cells MCF-12F) were used; in all cases, cells were exposed to different CuONPs concentrations. When exposed to the MIC for *Candida* (35.5 μg/mL), they show up to 80% cell viability, and then CuONPs can be considered non-toxic. However, fibroblasts were most susceptible at the concentration of 42.1 μg/mL with cell viability of about 50%. Breast cells had better viability since no toxic effects were detected at any of the tested concentrations. For cytotoxicity assays using nanoparticles, regularly breast cancer cells are used. However, it has already been shown that cancer lines such as MCF-7 can be induced apoptosis with nanoparticles, and the same nanomaterial does not present any cytotoxic effect in non-cancer lines such as MCF-12F and MCF-10A [46,47]. In a recent study, the fibroblasts exposed to biosynthesized copper nanoparticles displayed uniform growth and normal morphology, reflecting good biocompatibility [48]. However, the cell viability of fibroblasts exposed to 30 μg/mL of chemically synthesized copper nanoparticles was less than 60%. In order to reduce the levels of toxicity, the authors coated with starch the nanoparticles, and cell viability in fibroblasts increased by up to 75% [49]. This demonstrates that using biological material for nanoparticle synthesis can increase cell biocompatibility.

Another important assay for nanoparticles with high potential in biomedicine is the hemocompatibility test since, eventually, nanoparticles could enter the bloodstream. ISO 10993-4:2002 indicates that products with a hemolysis percentage of less than 5% can be considered hemocompatible. The hemolysis test was carried out at different concentrations of CuONPs and indicated hemocompatibility in most concentrations tested; also, cells exposed to the MIC (35.5 µg/mL) showed a low percentage of hemolysis (3.6%). The hemolysis assay in biologically synthesized CuONPs using extracts from the leaf of *Cassia occidentalis* had a hemolysis of 4.6%, similar to that reported in the present study [50]. These results indicate that CuONPs are hemocompatible at certain concentrations and have a high potential to be used for the elaboration of an antifungal topic treatment.

## 5. Conclusions

The biological synthesis of CuONPs was carried out with the supernatant of *Trichoderma* sp. in a simple and reproducible way. Small polydisperse nanoparticles were obtained (5.8 nm), and they display excellent antifungal activity against *C. albicans* with a MIC of 35.5 μg/mL. The CuONPs showed internalization and production of ROS in *C. albicans* cells. In addition, a significant result was the biocompatibility with different mammalian cell lines and the hemocompatibility of nanoparticles, having a hemolysis percentage below 5%, demonstrating their low toxic level with human cells. Therefore, the present study demonstrates that the biosynthesized CuONPs can be used as antifungal agents due to their high effectiveness and low cytotoxic level.

## Data Availability

Not applicable.
